# A One-Year Wastewater-Based Surveillance Study of the Main Human Respiratory Viruses in a Middle-Size Spanish City During the COVID-19 Pandemic Period

**DOI:** 10.3390/microorganisms14010151

**Published:** 2026-01-09

**Authors:** Lorena Casado-Martín, Marta Hernández, María José González-Peña, Mariana Alves-Elois, Nadine Yeramian, Gislaine Fongaro, José María Eiros, David Rodríguez-Lázaro

**Affiliations:** 1 Microbiology Area, Faculty of Sciences, University of Burgos, Plaza Misael Bañuelos s/n, 09001 Burgos, Spain; lcasado@ubu.es (L.C.-M.); maleves@ubu.es (M.A.-E.); nyeramian@ubu.es (N.Y.); 2 Centre for Emerging Pathogens and Global Health, University of Burgos, 09001 Burgos, Spain; 3 Microbiology Area, Faculty of Medicine, University of Valladolid, 47002 Valladolid, Spain; marta.hernandez.perez@uva.es (M.H.); jmeiros@uva.es (J.M.E.); 4 AquaVall, ETAP Las Eras, 47009 Valladolid, Spain; innovacion@aquavall.es; 5 Laboratory of Applied Virology, Department of Microbiology, Immunology, and Parasitology, Federal University of Santa Catarina, Florianópolis 88040-900, Brazil; gislainefongaro@gmail.com

**Keywords:** SARS-CoV-2, influenza viruses, respiratory syncytial virus, seasonality, prevalence, wastewater epidemiology, surveillance, one health

## Abstract

Respiratory infections are a major public health threat. Significant global mortality is caused by influenza viruses, the new SARS-CoV-2 virus, and the Respiratory Syncytial Viruses (RSVs). Wastewater-based epidemiology (WBE) has recently emerged as a valuable tool for monitoring these pathogens, providing insights into their evolution, transmission patterns, and co-circulation within populations. This study aimed to track influenza viruses (A and B), the new SARS-CoV-2 virus, and the Respiratory Syncytial Viruses (RSVs) (type A and B) during the pandemic period (from October 2020 to October 2021) in a middle-size Spanish city (Valladolid) and its surrounding areas. Viral concentration was performed using an aluminum-based precipitation method, followed by RNA extraction and RT-qPCR quantification targeting the N1 and N2 regions of the SARS-CoV-2 nucleocapsid gene, the N gene for both RSV-A and RSV-B, and the M and non-structural protein genes for influenza A and B, respectively. The results demonstrated the utility of WBE in predicting increases in clinical cases of SARS-CoV-2, as evidenced by a high correlation (r > 0.5). For RSV-A, the findings aligned with previous studies. Interestingly, particularly considering the length and period of analysis, influenza A, influenza B, and RSV-B viruses were not observed during the study period. In addition, the prevalence of RSV-A decreased during the SARS-CoV-2 pandemic, likely due to the implementation of non-pharmaceutical interventions. In conclusion, this study reaffirms that WBE provides critical epidemiological insights, complements clinical surveillance, and supports public health authorities in making informed and timely decisions.

## 1. Introduction

Respiratory infections, particularly lower respiratory infections (LRIs), are considered one of the eleven major public health threats declared by the Institute for Health Metrics and Evaluation in 2022 [1]. LRIs account for a substantial number of clinical cases and deaths annually; 489 million cases were reported globally, resulting in 2.5 million deaths, before the COVID-19 pandemic [2], with the highest burden observed in the Southern Hemisphere [3]. Before the emergence of SARS-CoV-2, the three primary etiological LRI agents responsible for the highest mortality rates were *Streptococcus pneumoniae*, influenza viruses, and the Respiratory Syncytial Viruses (RSVs) [4]. The clinical cases and mortality rates attributed to SARS-CoV-2 over the past few years have led to its mandatory inclusion in the list.

Wastewater-based epidemiology (WBE) has traditionally been used to monitor enteric viruses, including poliovirus, noroviruses, human astroviruses, rotaviruses, and hepatitis A and E viruses [5,6,7]. Historically, it was believed that enveloped viruses, including coronaviruses, Ebola virus, or influenza viruses, were not associated with fecal–oral transmission in humans due to their perceived susceptibility to degradation. Consequently, most WBE studies have focused on monitoring pathogens transmitted via the fecal–oral route, rather than those transmitted via respiratory, sexual, or vector-borne routes [8]. However, during the 2003 SARS-CoV-1 outbreak, evidence emerged linking SARS infections to viral detection in sewage systems, potentially associated with fecal aerosolization [9]. This finding prompted consideration of WBE as a tool for tracking a broader range of viruses, including enveloped and respiratory pathogens [10]. The utility of WBE gained renewed interest during the SARS-CoV-2 pandemic (2020–2023). Although its infection primarily manifests as respiratory symptoms, a significant proportion of infected individuals also exhibit gastrointestinal symptoms, accompanied by viral shedding in feces, saliva, or urine [11,12]. As a result, they enter the wastewater system, and monitoring this type of sample has become a valuable approach for tracking these pathogens and gaining insights into their evolution and transmission patterns within a population [13].

The use of WBE to monitor respiratory viruses, including SARS-CoV-2, influenza viruses, and the RSVs, can enhance our understanding of these pathogens while optimizing surveillance methodologies. Influenza viruses and RSVs are traditionally monitored in clinical settings, where only symptomatic individuals are tested. Notably, for RSVs, the most vulnerable populations—infants and elderly individuals—may wear diapers, leading to reduced viral detection in wastewater due to limited excretion [14]. Nevertheless, WBE can capture data from both symptomatic and asymptomatic individuals, including those not clinically monitored, who shed viral particles into the sewage system. During the COVID-19 pandemic, overwhelmed healthcare systems prioritized saving lives, focusing solely on patients who had tested clinically positive. Consequently, complementing clinical surveillance with WBE to track the prevalence of influenza viruses and RSVs offers a valuable approach. Positive correlations between wastewater concentrations of influenza A virus and RSVs and clinically confirmed cases was previously observed [15,16,17]. However, only a limited number of studies have monitored the prevalence of viral subtypes through WBE, despite the relevance of such data for public health authorities in implementing targeted control measures. In addition, most studies conducted in Spain on the circulation of respiratory viruses during the COVID-19 pandemic have focused on large metropolitan areas, such as Barcelona or Valencia [18,19]. As a result, there is a relative lack of evidence from medium-sized cities and from analyses conducted at a sub-local level, limiting the understanding of local variability in viral circulation. In this context, the present study provides a more granular and holistic perspective on respiratory virus transmission by analyzing different areas. This sub-local approach is particularly relevant for public health practice, as it enables the identification of local differences in disease burden and supports more targeted and context-specific decision-making, contributing to more efficient surveillance and prevention.

This paper describes a monitoring program for key respiratory viruses, including two target genes (N1 and N2) for SARS-CoV-2, influenza virus subtypes A and B (Inf A and Inf B), and RSV subtypes A and B (RSV-A and RSV-B), all of which pose a significant public health threat. The objectives of this work are threefold: to investigate the evolution and prevalence of SARS-CoV-2 during the pandemic period (October 2020 to October 2021) in the province of Valladolid, Spain; to correlate meticulously tracked local clinical cases with viral concentrations in the sewage system; and to evaluate the behavior of other respiratory viruses during the same period. Moreover, we aim to assess whether government measures implemented to curb the COVID-19 pandemic influenced the transmission dynamics of these pathogens.

## 2. Materials and Methods

### 2.1. Sampling Collection and Sampling Areas

Wastewater samples were collected biweekly from October 2020 to October 2021 (n = 25) at a wastewater treatment plant (WWTP) located in Valladolid, a region in Castilla y León, Spain, which serves approximately 350,000 inhabitants according to WWTP service data. Specifically, at each sampling time point, six wastewater inlet samples were collected at a single wastewater treatment plant, corresponding to inflows from six different areas of the province of Valladolid (Table 1), resulting in a total of one hundred fifty samples analyzed. The prevalence of SARS-CoV-2 was monitored twice a month during the described period, while subtypes of influenza virus and Respiratory Syncytial Virus were analyzed monthly during the same period (n = 78).

For each sample, one liter of wastewater was collected using sterile, thiosulphate-free PET containers (VWR^®^, Avantor, Radnor, PA, USA) during early-morning monitoring hours (7–10 a.m.). Immediately after collection, samples were transported on ice to the laboratory, stored at 4 °C, and processed within the first twelve hours to ensure sample integrity.

### 2.2. Viral Concentration Method and Nucleic Acid Extraction

A total of 200 mL of wastewater were transferred to a sterilized 250 mL PPCO centrifuge bottle (Thermo Fisher Scientific Nalgene Products, Waltham, MA, USA). Each sample was spiked with 100 µL of a 1:100 dilution of Mengovirus vMC_0_ (CECT, Burjassot, Spain) as a process control. Viral concentration was then performed using an aluminum-based adsorption–precipitation method, as described by Randazzo [20]. The spiked samples were adjusted to pH 6.0, followed by adding 1 part 0.9 N AlCl_3_ solution (Acros organics, Geel, Belgium) per 100 parts of sample. The pH was readjusted to 6.0, and the mixture was agitated using an orbital shaker at 150 rpm for 15 min at room temperature. Viral particles were subsequently concentrated via centrifugation at 1900× *g* for 30 min. The resulting pellets were resuspended in 10 mL of beef extract (3%, pH 7.4) (Lab-Lemco Powder, Oxoid, Thermo Fisher Scientific, Waltham, MA, USA) and transferred to 50 mL polypropylene centrifuge tubes (Corning, Corning, NY, USA). The suspension was shaken at 150 rpm for 10 min, and viral concentrates were recovered by centrifugation at 1900× *g* for 30 min. The final pellet was resuspended in 1 mL of phosphate-buffered saline (PBS) (Thermo Fisher Scientific, Waltham, MA, USA).

To ensure process reliability, three process controls were included for each sampling date. A sample process control (SPC) consisting of 200 mL of autoclaved tap water inoculated with the same amount of Mengovirus as the samples and processed identically was employed to assess the efficiency and accuracy of the methodology. A negative sample process control (NSPC) formed of 200 mL of the same SPC water and processed similarly to the samples without adding Mengovirus was employed to detect potential cross-contamination during processing. An extraction control was obtained with 3 mL of the same sterilized tap water and spiked with the same amount of Mengovirus as the samples, mimicking the approximate final viral concentrate volume, enabling us to assess the efficiency of the nucleic acid extraction step.

Nucleic acid extraction from the viral concentrates was conducted using the QIAmp Viral RNA Mini Kit (Qiagen, Hilden, Germany), according to the manufacturer’s instructions. A total of 150 µL of viral concentrate was used for the extraction.

### 2.3. Respiratory Virus Quantification by RT-qPCR

SARS-CoV-2 quantification was performed using a one-step RT-qPCR assay targeting the N1 and N2 regions of the nucleocapsid gene, as described by the Centers for Disease Control and Prevention [21]. For influenza A and B viruses, the targets were M and non-structural protein genes, and RT-qPCR primers, probes, and thermocycling conditions followed the protocol established by Sanghavi [22], with slight modification comprising a uniplex reaction of 40 cycles (instead of 45). For both RSV subtypes, the N gene was targeted [23]. Details on primers and probes are provided in the Supplementary Materials (Table S1).

Respiratory virus concentrations were determined through one-step RT-qPCR reactions using the TaqMan Fast Virus 1-Step Master Mix (Applied Biosystems, Waltham, MA, USA) in a final volume of 10 µL. Each reaction contained 2.5 µL of either undiluted, 10-fold diluted, or synthetic RNA. All experiments were conducted on a QuantStudio5 thermocycler (Applied Biosystems, Waltham, MA, USA). The RT-qPCR conditions followed those described in the original protocols, except for the reverse transcription step, which was shortened according to the TaqMan Fast Virus 1-Step Master Mix instructions. This modification reduced the total reaction time without compromising assay sensitivity [24].

Each RT-qPCR assay was performed in duplicate for both undiluted RNA and a 10-fold dilution to detect potential inhibition. Non-template controls, including the NSPC, SPC, and extraction control, were also included in duplicate assays on the same plate. Standard curves for each virus and assay were generated in every experiment to account for the intrinsic variability of RT-qPCR and to ensure more reliable quantification. These curves were constructed using 10-fold serial dilutions of commercially available quantitative synthetic RNA (Supplementary Table S2).

Viral titers were estimated by converting cycle threshold (Cts) values ≤ 40 obtained with QuantStudio^TM^ Design & Analysis software (v1.5.1) into genomic copies per liter (gc/L) using the corresponding standard curves and dilution factors. For consistency and comparability, a fixed threshold was manually set close to the software’s recommended values: 0.05 for SARS-CoV-2 and influenza viruses, and 0.04 for RSVs.

Inhibition was assessed by comparing the average viral titers of undiluted and 10-fold diluted RNA samples. Inhibition was considered significant if the difference in viral titers exceeded 0.5 log_10_. In such cases, viral concentrations were calculated from the 10-fold-diluted RNA values to ensure accurate quantification.

### 2.4. Data Validation, Normalization, and Statistical Analysis

All the assays underwent rigorous validation through different steps. First, negative controls were examined to confirm the absence of amplification. In such cases, the RT-qPCR assay was repeated. Second, standard curves were generated for each assay, and their quality parameters (e.g., efficiency, r-adjustment) were evaluated to ensure that they met acceptable thresholds, accounting for the intrinsic variability between experiments. Upon validation of the results, quantification was performed as described in the previous Section, followed by data normalization.

In order to compare prevalence across areas with bearing population densities and time periods, normalization is an essential component of WBE studies [9,25]. In this study, viral concentration data were normalized per 100,000 inhabitants using population size estimates provided by the wastewater treatment plant (WWTP) (Table 1). For each area with little tourism activity, this *de facto* population normalization method is considered adequate and suitable, particularly in situations when data on other factors such as hydraulic and/or chemical data are not available [26,27].

Clinical case data were obtained from the Junta de Castilla y León databases [28], including all individuals with laboratory-confirmed COVID-19 during the study period (RT-qPCR, rapid test, etc.). To assess the correlation between viral concentration levels and clinical cases of SARS-CoV-2, Spearman’s rank correlation analysis was performed using R (version 4.4.0, 2024-04-24 ucrt). The correlation coefficient was calculated using mean genomic copies per liter gc/L aggregated across the six monitored areas.

## 3. Results

### 3.1. One-Year Evolution of SARS-CoV-2 and Comparative Analysis of N1 and N2 Gene Targets

**Figure 1:**
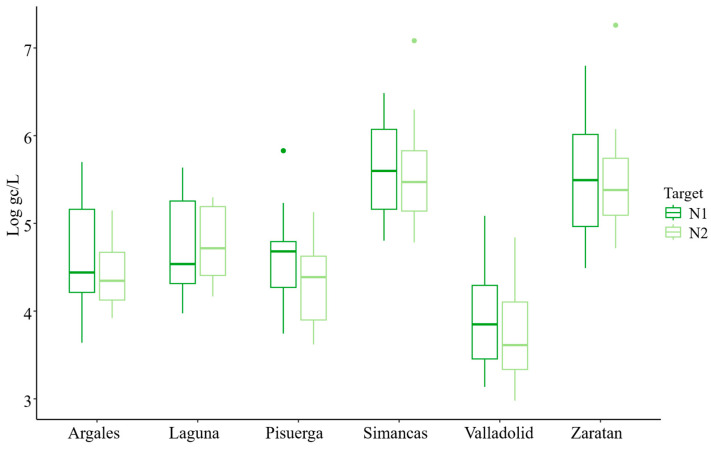
Mean Evolution of SARS-CoV-2 N1 and N2 targets across six sampling areas (Log gc/L; Logarithmic Units of genome copies per liter), normalized per 100,000 inhabitants.

Influent samples were collected biweekly from October 2020 to October 2021 from a wastewater treatment plant (WWTP) located in Valladolid, Spain, and analyzed using a one-step RT-qPCR assay to monitor the presence of SARS-CoV-2. The temporal dynamics of SARS-CoV-2 were assessed by targeting two regions of the nucleocapsid gene, N1 and N2. The virus had been detected in the population only a few months before the initiation of this study. Throughout the study period, SARS-CoV-2 was consistently detected in wastewater, indicating its continuous prevalence (Figure 1).

**Figure 2:**
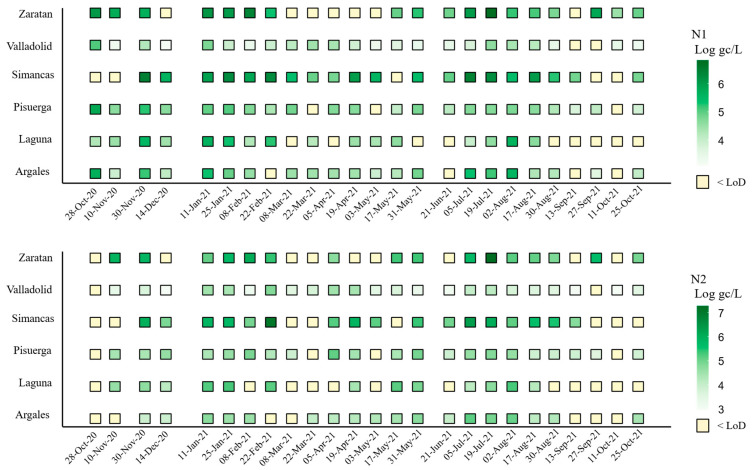
Viral load of SARS-CoV-2 N1 and N2 targets (Log gc/L, normalized per 100,000 inhabitants) by area and sampling date. Note: N2 was not analyzed on 28 October 2020.

The average concentration during the study period was 4.79 ± 0.07 and 4.64 ± 0.07 Log gc/L per 100,000 inhabitants for N1 and N2, respectively (Table 2). The overall trends for both N1 and N2 targets were similar and closely aligned (Figure 1 and Figure 2). However, at the beginning of the study, the concentration of the N2 target was lower than that of N1. While the mean concentration of N1 was generally higher than that of N2, the maximum concentration was observed for the N2 target (7.26 Log gc/L) (Figure 2). Additionally, the percentage of samples with concentrations below the limit of detection (LoD) was greater for N2 (28%) than for N1 (20%). Boxplot analysis indicated greater variability in N1 concentrations (evidenced by longer boxes and whiskers) compared to N2, except in the Pisuerga region.

Significant increases in concentration were observed at several points during the study, with the most pronounced peaks occurring on 30 November 2020, 11 January 2021, and 19 July 2021 (Figure 2). Additional secondary peaks, characterized by lower concentration levels, were detected in February, April, May, and September 2021 (Figure 2).

### 3.2. One-Year Evolution of SARS-CoV-2 by Area

Comparison across the six areas studied showed that Simancas exhibited the highest mean SARS-CoV-2 concentrations for both targets, with values of 5.53 and 5.50 Log gc/L per 100,000 inhabitants for N1 and N2, respectively, followed by Zaratan and Laguna. In contrast, Valladolid had the lowest mean concentration, with 3.90 and 3.71 gc/L per 100,000 inhabitants for N1 and N2, respectively (Figure 2). This location also demonstrated the most homogeneous presence of SARS-Co-2, with only 8% of the samples having concentrations below the LoD (Figure 2), demonstrating a consistent viral incidence without notable fluctuation in concentration measures.

**Figure 3:**
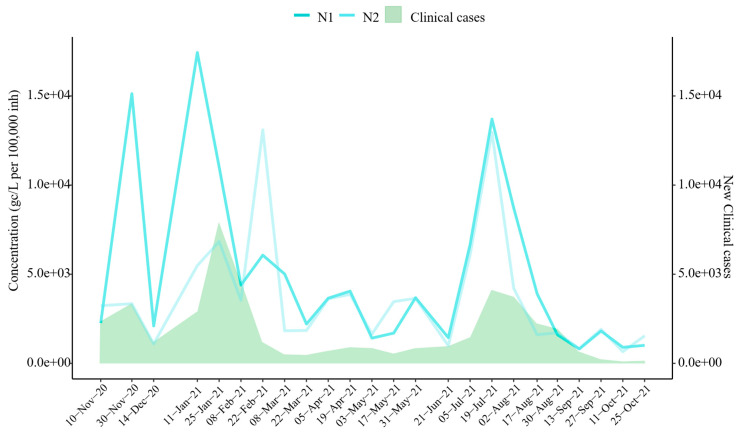
SARS-CoV-2 Target Trends *vs.* Clinical Cases. Evolution of SARS-CoV-2 targets (mean concentration across six sampling areas) and new clinical cases reported by the Junta de Castilla y León [28]. Note: N2 was not analyzed on 28 October 2020.

Zaratan exhibited the second-highest mean concentrations (Figure 2), despite experiencing low levels from March to May 2021 (Figure 3). The SARS-CoV-2 heatmap revealed that although Laguna had high mean concentration levels (Figure 2), it also displayed the highest proportion of samples with concentrations below the LoD, particularly for the N2 target (48%). This fact was especially evident across the final five sampling dates, from August to October 2021.

Regarding the negativity rates (samples with concentrations below the LoD), Laguna (36% for N1, 48% for N2), Zaratan (28% for N1, 36% for N2), and Simancas (20% for N1, 32% for N2) recorded the highest rates for both targets.

### 3.3. Clinical Cases and SARS-CoV-2 Correlation

The results reveal a high temporal correlation between wastewater SARS-CoV-2 concentrations and clinical case trends during the study period (Figure 3). Spearman’s correlation analysis confirmed significant associations between wastewater viral concentrations and clinical case numbers, with correlation coefficients of r = 0.657 for N1 and r = 0.544 for N2 (*p*-values < 0.05). Notably, the significant increase observed in November 2020 was detected despite the slight decrease recorded on 10 November 2020. Similarly, the surge in January 2021 was identified earlier in wastewater data than in clinical case reports, as the N1 target indicated a significant increase on 11 January 2021. Furthermore, the sharp increase in viral concentrations observed in July 2021 closely coincided with clinical case data. Smaller clinical waves, occurring in April and May 2021, were also detected in wastewater surveillance. However, increases in SARS-CoV-2 concentrations in February and September 2021 were identified through wastewater analysis but were not reflected or reported in clinical data.

### 3.4. Evolution of Other Respiratory Viruses Studied

**Figure 4:**
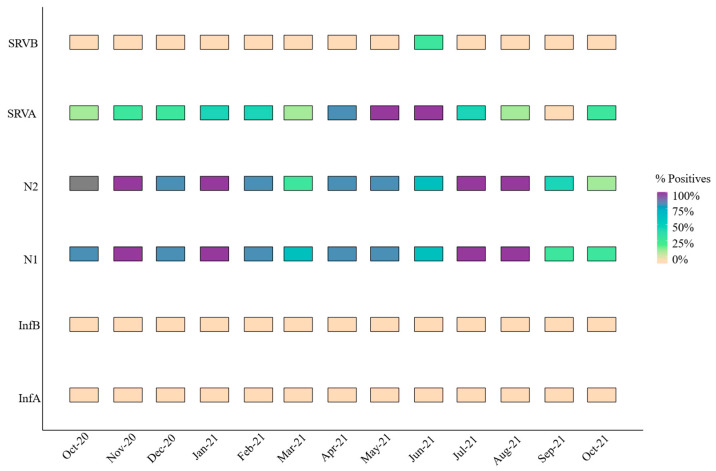
Monthly positivity rate (%) for all respiratory viruses or targets.

Figure 4 summarizes the positivity rates (%) during the study. Except for N2 in October 2020, when it was not examined, SARS-CoV-2 was continuously present over the sample period, but influenza A and B viruses were absent and RSV-B was only present for one month. SARS-CoV-2 positivity rates varied across its two targets, ranging from 25 to 100%. For instance, N2 had greater positivity rates in March and October of 2021, but N2 had lower values in September compared to N1. RSV-B was not detected in most samples, except on 21 June 2021, when the positivity rate reached 33% with only two positive samples. Specifically, one of four replicates tested positive in Valladolid, with a concentration level of 3.54 Log gc/L (not normalized per 100,000 inhabitants). In Zaratan, all undiluted samples were amplified, yielding a concentration of 3.97 Log gc/L.

In contrast, RSV-A exhibited the highest positivity rate among the respiratory viruses studied, excluding SARS-CoV-2. This pathogen was particularly prevalent from April to June 2021, with a marked increase during these months (Figure 4). Simancas and Zaratan recorded the highest mean concentration levels at 5.81 ± 0.02 and 5.38 ± 0.14 Log gc/L, respectively. These findings highlight a dichotomy: RSV-A was either undetectable or highly prevalent.

**Figure 5:**
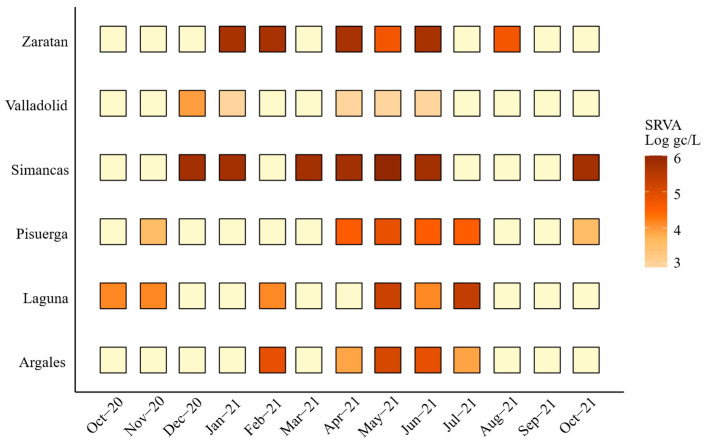
Monthly viral load of Respiratory Syncytial Virus A (RSV-A) expressed as Log gc/L per 100,000 inhabitants by area. Light yellow indicates values below 3 Log gc/L.

Additionally, Figure 5 shows how Simancas, Zaratan, and Valladolid exhibited a rise in RSV-A concentrations during December–January and January–February. Interestingly, a peak in SARS-CoV-2 was also observed in December 2020 in the wastewater samples and a peak in clinical cases was subsequently seen in January 2021 (Figure 3). Towards the end of 2021, another peak was observed in Simancas and Pisuerga. However, the overall RSV-A positivity rate was 44.9%, substantially lower than that for SARS-CoV-2 targets N1 (80%) and N2 (75%).

## 4. Discussion

This study analyzed the presence and evolution of SARS-CoV-2 throughout different areas within a single region, as well as the correlation between viral concentrations detected in wastewater and clinical cases meticulously recorded at that time. Additionally, the prevalence and co-circulation of other important respiratory viruses were also monitored.

SARS-CoV-2 was detected throughout the study period, exhibiting a consistent prevalence, despite local-level variations. The average viral concentration was slightly above 5 Log gc/L, consistent with similar findings from other regions in Spain [18,29]. Notably, the N1 region was quantified more effectively than its homologous N2. Although both targets should theoretically exhibit similar concentrations, the N2 target had a higher percentage of samples below the detection limit. This discrepancy may be attributed to a lower sensitivity of the RT-qPCR system for N2. This finding highlights how crucial it is to use a variety of genetic targets to gain a more precise estimate of a virus concentration, especially for novel pathogens [30].

Interestingly, some regions in this study, namely Laguna, Zaratan, and Simancas, displayed high average virus concentrations along with significant amounts of undetectable quantities. This paradox could be explained by several reasons. Firstly, in smaller communities, viral peaks are brief and timely concentrated, causing intermittent concentrations below detection limits, whereas in larger populations, the virus is present more steadily. Secondly, this may be exacerbated by the fact that mean values are calculated without taking into consideration negative (undetectable) values.

In contrast, Valladolid, with its larger population, exhibited more stable and homogeneous viral concentrations when normalized to 100,000 inhabitants. There are two potential causes of this effect. First, the size of the population affects the normalization technique that is employed, resulting in varying effects. Smaller populations produce higher normalized values when the virus concentration is the same, while bigger populations lessen this effect. Second, the higher water flow may have a diluting effect even if Valladolid has a larger population and a higher fecal deposition index.

According to other studies [18,31,32], viral concentration peaks in November 2020 and January, April, and July 2021 corresponded with national clinical case waves, particularly the so-called second to fifth waves. These findings reinforce the utility of wastewater-based epidemiology (WBE) as a tool for detecting increases in viral presence within populations, a key reason for its adoption [33]. However, as reported in the literature [34,35], the expected anticipated earlier detection trends in wastewater were less noticeable in this study, likely due to the biweekly sampling frequency, which may have hindered the detection of early trends. There was a positive and comparatively strong association between viral concentration and clinical cases, with better results for the N1 gene (r ~ 0.7). This value is slightly lower than in other published studies [], perhaps as a result of the shorter study duration.

In line with national data from Spain’s Acute Respiratory Infection Surveillance Report for the 2020–2021 season [36], which reported a significant decrease in influenza activity, SARS-CoV-2 was detected across all sampling dates, while no influenza viruses were identified. Respiratory Syncytial Virus A (RSV-A) and B (RSV-B) were detected in 44.87% (35/78) and 2.56% (2/78) of the samples, respectively. The RSV-A positivity rate fell within the lower range observed in prior seasons (30–80%) [37], reflecting a worldwide decline in RSV-A circulation as a result of non-pharmaceutical interventions (NPIs) such as mask-wearing and social distancing that were put in place to lessen the spread of SARS-CoV-2. These NPIs also seem to have an impact on the transmission of the majority of respiratory viruses [38,39]. On a worldwide scale, this dramatic drop in RSV-A clinical cases has been noted in nations including the EEUU, South Africa, France, Alaska, Australia, and Japan [34,,36,37,38,39].

Despite this overall decline, a peak in RSV-A was observed in spring 2021, particularly in wastewater data, which came before the clinical peak. This trend was consistent with national clinical data, further supporting WBE as an early indicator of clinical outbreaks for various respiratory viruses including RSV. This occurrence of sickness peaks in late spring is noteworthy because the infection usually circulates across Europe during the winter months [40]. The unusual increase during the spring could be attributed to factors such as relaxed social restrictions, increased outdoor activity, and reduced population immunity due to decreased exposure during the pandemic [41,42]. However, it is important to note that spring peaks have also been sporadically observed in other Mediterranean countries like Greece [43,44].

An RSV-A peak detected in December 2020 in Simancas and Pisuerga [36,37] underscores the utility of WBE for tracking viral circulation and identifying outbreaks, consistent with evidence of strong correlations between wastewater RSV detection and clinical cases [15,27,45]. It is also noteworthy that RSV-A and RSV-B usually co-circulate [45], whereas during the COVID-19 pandemic, RSV-A was the dominant lineage [39], consistent with our findings.

The lack of influenza viruses in wastewater is consistent with national data [36], which reported a decline of more than 99% compared to previous seasons. This result is also consistent with international observations made by the US and Japan, among other countries [36,39,46]. Wastewater samples did not contain influenza, despite the fact that it was discovered in some European regions in late September 2021 [33,36,47]. This result might be explained by the fact that influenza requires a higher clinical incidence for detection in wastewater.

## 5. Conclusions

This study confirms that WBE provides critical epidemiological insights that enable the monitoring of infectious organisms both geographically and temporally. Even in areas with relatively small populations, SARS-CoV-2 results via WBE indicate a strong correlation between quantified virus concentrations and clinical cases. Furthermore, this study, like many others worldwide, highlights a generalized decrease in the circulation of respiratory viruses, excluding SARS-CoV-2. This phenomenon is generally attributed to hygiene measures and social distancing protocols implemented to contain the ongoing pandemic at the time.

Finally, the overwhelming strain on healthcare systems during the pandemic, which prioritized saving lives, hindered clinical monitoring of influenza and RSV cases. Consequently, this situation may have led to an underestimation of the prevalence of influenza and RSVs. This phenomenon emphasizes the significance of wastewater surveillance, which has been shown to be useful for monitoring a variety of infections. In retrospect, WBE provides a more accurate picture of the population’s health status concerning infectious agents when combined with clinical data. This allows public health authorities to make prompt, well-informed choices, especially during pandemic or epidemic outbreaks.

## Figures and Tables

**Table 1:** Demographical data per area (provided by WWTP service).

Area	Inhabitants
Valladolid	350,000
Zaratan	6400
Simancas	5500
Argales	40,000
Laguna	22,700
Pisuerga	80,000

**Table 2:** Statistical parameters of N1 and N2 SARS-CoV-2 targets.

	N1	N2
Mean	4.79	4.64
St. Error	0.07	0.07
Min	3.14	2.98
Max	6.80	7.26

## Data Availability

The raw data supporting the conclusions of this article will be made available by the authors on request.

## Supplementary Materials

The following supporting information can be downloaded at https://www.mdpi.com/article/10.3390/microorganisms14010151/s1: Table S1: Oligos and TaqMan^TM^ probes for respiratory viruses studied; Table S2: Standard material references.
