# Supplementary material for: A One-Year Wastewater-Based Surveillance Study of the Main Human Respiratory Viruses in a Middle-Size Spanish City During the COVID-19 Pandemic Period
Source: Microorganisms. 2026 Jan 9;14(1):151. doi: 10.3390/microorganisms14010151 (PMC12843686; doi:10.3390/microorganisms14010151)
Supplement: Supplementary file 1 [file microorganisms-14-00151-s001.zip › microorganisms-4016552-supplementary.pdf]

Supplementary material

Virus	Primer name	Primer/probe sequence 3'-5'	Reference
**SARS-CoV-2 N1**	2019-nCoV_N1-F	GACCCCAAAATCAGCGAAAT	(CDC, 2020)
	2019-nCoV_N1-R	TCTGGTTACTGCCAGTTGAATCTG	
	2019-nCoV_N1-P	FAM-ACCCCGCATTACGTTTGGTGGACC-BHQI	
**SARS-CoV-2 N2**	2019-nCoV_N2-F	TTACAAACATTGGCCGCAAA	(CDC, 2020)
	2019-nCoV_N2-R	GCGCGACATTCCGAAGAA	
	2019-nCoV_N2-P	FAM-ACAATTTGCCCCCAGCGCTTCAG-BHQ1	
**Influenza A**	FluA For	CAT GGA RTG GCT AAA GAC AAG ACC	(Sanghavi et al., 2012)
	FluA Rev	AGG GCA TTT TGG ACA AAK CGT CTA	
	FluA P	FAM-TGC AGT CCT CGC TCA CTG GGC ACG-BHQ1	
**Influenza B**	InfB For	TCC TCA AYT CAC TCT TCG AGC G	(Sanghavi et al., 2012)
	InfB Rev	CGG TGC TCT TGA CCA AAT TGG	
	FluB P	FAM-CCA ATT CGA GCA GCT GAA ACT GCG GTG-BHQ1	
**RSVA**	A21	GCTCTTAGCAAAGTCAAGTTGAATGA	(de-Paris et al., 2012)
	RSV Compl	AACATGCCACATAACTTATTGAT	
	APB48	FAM-ACACTCAACAAAGATCAACTTCTGTCATCCAGC-BHQ1	
**RSVB**	B17	GATGGCTCTTAGCAAAGTCAAGTTAA	(de-Paris et al., 2012)
	B120	TGTCAATATTATCTCCTGTACTACGTTGAA	
	BPB45	FAM-TGATACATTAAATAAGGATCAGCTGCTGTCATCCA-BHQ1	

Table S1: Oligos and TaqMan™ probes for respiratory viruses studied.

Virus	Standard Reference
**SARS-CoV-2 (N1 and N2)**	Twist Synthetic SARS-CoV-2 RNA Control 1-MT007544.1
**Influenza A virus**	Quantitative Genomic RNA from Influenza A virus H1N1 strain A/California/07/2009 (H1N1) pdm09 (ATCC VR-1894DQ™)
**Influenza B virus**	Quantitative Genomic RNA from Influenza B virus (Yamagata lineage) strain B/Wisconsin/1/2010 (ATCC VR-1883DQ™)
**RSVA**	Quantitative Genomic RNA from Human respiratory syncytial virus strain A2 (ATCC VR-1540DQ™)
**RSVB**	Quantitative Genomic RNA from Human respiratory syncytial virus strain 9320 (ATCC VR-955DQ™)

Table S2: Exogenous standard material references.
